# An Abnormal Pulp Canal

**Published:** 1892-10

**Authors:** C. F. W. Bodecker

**Affiliations:** New York City. Professor of Embryology, University of Buffalo, Dental Department.


ARTICLE III.

### AN ABNORMAL PULP CANAL.

BY C. F. W. BODECKER, M. D. S.; D. D. S.; NEW YORK CITY.
PROFESSOR OF EMBRYOLOGY, UNIVERSITY OF BUFFALO, DENTAL DEPARTMENT.

Mrs. W., aged about thirty-five years, with a good constitution, was about two years ago suffering from severe pulpits of the left upper second bicuspid. The tooth, five years previously had been filled with oxyphosphate cement. The filling was now taken out and an attempt made to devitalize the pulp, but the application had to be removed, as the pain became too intense for the patient to bear. The pulp was then treated for a few days, and when the inflammation had subsided it was capped and the cavity filled with gutta-percha.

The tooth remaining very sensitive to thermal changes, after about one year the patient decided to have it treated again. The filling and cap were removed, the pulp being found apparently in a normal condition, but as the patient insisted upon having either the pulp or the whole tooth taken out, I decided to make an application of the chloride of methyl spray and extripate the pulp.

A microscopical examination of the coronal portion of the pulp showed that, with the exception of a few spots having calcareous depositions, it had been in a normal condition. The application of the methylic spray was very painful, but the removal of the pulp gave the patient but little pain. The canal was enlarged with Morey drills, disinfected with a solution of peroxyde of hydrogen, dehydrated with absolute alcohol and hot air, and immediately filled with chloro-percha. The introduction of the filling material into the canal produced intense pain. After repeated applications of counter irritants and ice to the gum above the affected tooth, the pain gradually subsided.

Three days after this, the patient became intermittently afflicted with neuralgic pains of that side, affecting the eye, the ear and the neck. The longer the duration of these paroxysms, which lasted from one to three hours, the more severe became the pain. The tooth in this stage exhibited no signs of pericemental inflammation, nor was there any pain produced by pressure upon the gum surrounding it. I then consulted with her family physician, who prescribed tonics and anti-neuralgic remedies, but without the slightest relief.

The seventh day after the canal had been filled the tooth became sore to the touch, when I concluded to remove the filling from the canal. This produced instant relief. In removing the filling, I noticed that as soon as the instrument was pushed along the distal portion of the canal, about the middle of the root, the patient experienced great pain. It appeared to me that either a small portion of pulp must have been left in this place, or that there was an enlargement of a dentinal fibre. I made an application of a saturated solution of hydrochlorate of cocaine, and with the largest Morey crown drill reamed out the canal, which was found to be perfectly straight. As the instrument reached about the middle of the root it produced very severe pain, but as soon as the drill had passed this sensitive point it gave no pain whatever.

Upon the removal of the drill I found some blood in the pulp canal, which was thoroughly rinsed with water and dried, when an application of pure carbolic acid upon cotton was inserted.

A thorough examination the next day proved that the pulp gave off a branch running at right angles toward the distal portion of the root. This branch was about as large as the main body of the pulp in that portion of the tooth, and was supplied with blood-vessels. I then concluded to coagulate a portion of this pulp tissue by successive applications of pure carbolic acid. As there is danger of pericemental irritation after repeated use of this remedy, I closed the apical foramen with a small piece of cotton saturated with melted paraffine.

After applying the carbolic acid for six days, I removed the paraffine from the apex of the canal, and filled this portion with gutta-percha. The remainder of the pulp cavity was filled temporarily with cotton, saturated with a fresh solution of iodoform in sulphuric ether, and the cavity was closed with gutta-percha, After two weeks the temporary filling and cotton were removed again, and tin foil was substituted. The tin was introduced into the canal by means of small burnishers in the engine, the upper portion being filled with gutta-percha. All the neuralgia has disappeared, and the tooth has given no trouble since.—*Dental Pract. and Advertiser.*

